# Botulism in waterfowl: case report in Argentina

**DOI:** 10.1186/s13028-025-00824-7

**Published:** 2025-08-07

**Authors:** María Florencia Ovelar, Germán José Cantón, Jorge Pablo García, María Belén Riccio, Alicia Raquel Rodríguez, María Isabel Farace, Ignacio Alvarez

**Affiliations:** 1 Instituto de Innovación para la Producción Agropecuaria y el Desarrollo Sostenible, (IPADSINTA Balcarce-CONICET), Ruta 226 km 73.5, Balcarce, 7620 Argentina; 2 Servicio de Diagnóstico Veterinario de Tandil, Centro de Investigación Veterinaria de Tandil (CIVETAN), Facultad de Ciencias Veterinarias, Universidad Nacional del Centro de la Provincia de Buenos Aires, C. Arroyo Seco SN, Tandil, B7000 Argentina; 3 Servicio de Bacteriología Sanitaria, Instituto Nacional de Enfermedades Infecciosas ANLIS Dr. Carlos Malbrán, Av. Vélez Sarsfield 563, Ciudad Autónoma de Buenos Aires, C1282 Argentina; 4 Department of Clinical Sciences, Swedish University of Agriculture Sciences, 8 Almas Allé, Uppsala, 75007 Sweden

**Keywords:** Avian disease, *Clostridium botulinum*, Neurotoxin, Type C toxin, Wild birds, Wildlife

## Abstract

**Introduction:**

Botulism, a severe neuroparalytic disease caused by the botulinum toxin produced by *Clostridium botulinum*, poses significant threats to wild birds. This study describes a natural outbreak of type C botulism in waterfowl in the surroundings of a lagoon in Saavedra, Buenos Aires province, Argentina, during January 2021. The outbreak, affecting approximately 300 birds, was attributed to environmental conditions that allowed the proliferation of *C. botulinum*. Clinical signs included progressive weakness, paresis, flaccid paralysis, difficulties in locomotion and swimming, “limbing neck”, and nictitating membrane protrusion. No gross lesions were observed during autopsies, but mild congestion, hemorrhage, and pulmonary edema were noted microscopically. Toxin type C was detected in feces, serum samples, and lagoon water, confirming the diagnosis. This is the first documented report of waterfowl botulism in central Argentina and highlights the impact that delayed detection can have on bird populations.

**Background:**

Botulism, a severe neuroparalytic disease caused by *Clostridium botulinum* neurotoxins, poses a significant risk to wild birds, especially waterfowl and their ecosystems. Recent trends show an increase in botulism outbreaks in wild birds, likely influenced by climate change impacting environmental factors. Unlike in humans, there is often a lack of regulation and surveillance of botulism in wild birds worldwide.

**Case presentation:**

In January 2021, an outbreak of neurological disease characterized by locomotion difficulties, led to the deaths of approximately 300 waterfowl. Results confirmed BoNTs type C establishing the cause of the mortality.

**Conclusions:**

This botulism outbreak underscores the critical need for early detection and intervention to prevent significant losses in wild bird populations.

**Supplementary Information:**

The online version contains supplementary material available at 10.1186/s13028-025-00824-7.

## Background

Botulism is a severe neuroparalytic and potentially lethal disease that can affect a wide range of animal species, including humans [1]. It is caused by botulinum neurotoxins (BoNTs), which are produced by the spore-forming bacterium *Clostridium botulinum*. Under conditions of anaerobiosis, temperatures between 25 and 40 °C and adequate nutrients, *C. botulinum* replicates in various terrestrial and aquatic environments [2–4].

Among vulnerable species, wild birds are one of the most commonly affected, with outbreaks associated with BoNT types C/D, C, and E. However, since discrimination between BoNT type C and the mosaic type C/D is not always performed in routine diagnostics, detections reported as type C may actually correspond to either type C or C/D [5–8]. Botulism is recognized as one of the most severe diseases affecting migratory birds and waterfowl, with significant impacts on species populations and ecosystems [1]. Botulism has been reported in at least 264 bird species, primarily waterbirds, with a particular high incidence in the *Anatidae* family [9]. The disease has been associated with the loss of 15% of the western population of American white pelicans (*Pelecanus erythrorhynchos*), the disappearance of great black-backed gulls (*Larus marinus*) from eastern Lake Ontario (Canada and USA), and a 7% decline in the population of black-faced spoonbills (*Platalea minor*) in Taiwan [10–12]. In Argentina, Rosciano et al. reported an outbreak affecting 2.5% of adult kelp gull (*Larus dominicanus*) population in colonies around lake Nahuel Huapi lake in Patagonia during the austral summer of 2020 [13].

Over the last decades, the impact of climate change on risk factors contributing to the replication of *C. botulinum* has become an important topic of discussion [2, 14]. Rising temperatures, altered precipitation patterns, and lower water levels during droughts can create anaerobic conditions favorable for the germination and replication of *C. botulinum*. However, although *C. botulinum* is known to produce BoNTs under laboratory conditions, direct evidence of toxin production in natural aquatic environments is still limited and largely inferential [2].

While humans are protected by strict regulatory controls and active disease monitoring, wildlife species often lack such regulation in several countries. This discrepancy is likely to be due to the perception of the authorities regarding the economic impact of wildlife diseases and the risk of transmission to humans. As a result, wild bird botulism is not a notifiable disease in many regions [1] This oversight can lead to significant underreporting and inadequate management of outbreaks, masking the full ecological impact and impeding effective response strategies [15].

This study aims to describe the first outbreak of botulism in waterfowl in the central region of Argentina, not only documenting epidemiologic, clinical, and pathological findings, but also highlighting the role of local community participation in outbreak detection and response.

## Case presentation

In January 2021, an unusual neurological disease and mortality event affecting approximately 300 waterfowl was registered in a lagoon located on a private farm in the Saavedra District, Buenos Aires Province, Argentina. The investigation of the case was requested 15 days after the landowner observed an abnormal number of waterfowl deaths in the lagoon. The most affected species (around 70%) was *Anas georgica* (yellow-billed pintail or “Pato maicero”), as well as *Fulica* sp. (“gallaretas”), *Plegadis chihi* (white-faced ibis) and *Cygnus melacoryphus* (black-necked swan). No signs of disease were found in other bird species identified in the lagoon, such as *Phoenicopterus chilensis* (Chilean flamingo) and *Ardea alba* (great egret). Affected birds presented progressive weakness, paresis, flaccid paralysis of skeletal muscles, difficulty in locomotion and swimming, limber neck, and protrusion of the nictitating membranes. During the 32 days before the outbreak investigation, the average daily temperature ranged from 16.7 °C to 31.2 °C, with maximum temperatures exceeding 30 °C on 22 of these days. Following the first carcass removal, performed during the field investigation, a progressive decrease in ambient temperature was recorded, with average daily temperatures falling below 25 °C in the following 20 days (Additional file 1). The lagoon, where the affected birds were found, was surrounded by cultivated fields covering an area of 270,281 m² and reaching a maximum depth of about 1.5 m. During the field investigation, water pH was measured using a colorimetric indicator strip, which indicated a pH of 9. Additionally, green algae were visibly present on the surface of the lagoon at the time of the investigation.

To investigate the cause of the outbreak, four yellow-billed pintails presenting clinical signs were autopsied. Samples of liver, kidney, lung, cecum and tonsils, small intestine, brain, spleen, gizzard, pancreas and heart were collected and fixed by immersion in 10% buffered formalin (pH of 7.2) for routine histopathological analysis. At the time of post-mortem examination, feces and blood were collected from two animals and kept at 4 °C. During the field investigation, 500 mL of lagoon water was collected in a sterile bottle from the lagoon coast. The water sample was kept at 4 °C and transported to the laboratory for bacteriological examination. Bacterial culture and toxin identification were conducted on collected samples. A total of 3 mL of water and feces were individually cultivated in BBL Cooked Meat Medium (Becton, Dickinson and Company, Franklin Lakes, NJ, USA) at 37 °C for 5 days under anaerobic conditions for *C. botulinum*. After the culture, samples were centrifuged, and the supernatant was filtered using 0.45 μm Millipore filter (Merck KGaA, Darmstadt, Germany). Serum and the previously described extract from water and feces were individually assessed for type C BoNT by inoculating albino mice (weighting 18–20 g) on a duplicate mouse bioassay, as previously described by Villar et al. [16]. Briefly, 0.5 mL of either serum or extract, was mixed with 0.1 mL of *C. botulinum* type C monovalent antitoxin (Institut Pasteur, Paris, France) and incubated at 37 °C for one hour before intraperitoneal inoculation into the mice. In parallel, another group of mice was intraperitoneal injected with the same inoculum but without the antitoxin.

No gross lesions were observed at necropsy of the birds. All four yellow-billed pintails autopsied showed digested food in the digestive tract. Microscopically, mild congestion and hemorrhage, as well as pulmonary edema (amorphous eosinophilic material in the parabronchi lumen) was observed in the lungs of birds #1, #2 and #3. Hepatic congestion was observed in bird #2. Furthermore, 90% of hepatocytes contained a fine brown granular material (hemosiderin). Moderate lymphocytic and heterophilic periportal infiltration was also observed in the liver of bird #2.

*Clostridium botulinum* type C was isolated from the lagoon water and feces of both sampled birds after enrichment culture. In the mouse bioassay, mice injected with the enriched extract and type C-specific neutralizing antitoxin survived, while those inoculated with the same extract without antitoxin died, indicating the presence of toxigenic *C. botulinum*. One serum sample tested negative, whereas the fecal extract from the same bird tested positive. History, clinical signs, pathological and bacteriological findings, including culture and mouse bioassays confirmed the first outbreak of BoNT type C in waterfowl in central region of Argentina.

Due to the suspicion of botulism during the investigation, carcasses were collected and burned daily on-site using open-air pyres. This intervention began on the day of sampling and continued for approximately 20 days. The method was selected due to the absence of specific national guidelines for carcass disposal in wildlife botulism outbreaks. Following the implementation of these measures, the number of carcasses found declined progressively, and mortality ceased completely 20 days after outbreak management actions were initiated [17].

## Discussion and conclusions

This case represents the first documented outbreak of avian botulism type C in waterfowl in the central region of Argentina, occurring in a lagoon where environmental conditions known to favor botulism were observed. Of particular relevance is the combination of high temperatures, shallow and alkaline water, the presence of algae and the accumulation of carcasses, all of which created optimal conditions for *C. botulinum* proliferation. In addition, the involvement of the landowner in reporting the event demonstrates the critical role of the community in the early detection of wildlife diseases in regions where formal surveillance systems are lacking. This underscores the need for improved multidisciplinary strategies that combine environmental monitoring, community engagement and rapid intervention in outbreak management.

The lack of previous reports of avian botulism in central Argentina could indicate either an actual absence of this condition in the region or inadequate disease surveillance. This incident raises the question if recent environmental changes may have triggered the first detectable outbreak in the area where the disease had previously gone unnoticed. In this case the number of dead birds was particularly high relative to the size of the lagoon and the typical waterfowl population present. There were no similar mortality events observed in recent years, which made this sudden mortality event particularly significant and encouraged the landowner to report the incident. The carcasses were first noticed 15 days before the investigation began, suggesting that the outbreak likely began earlier. Earlier detection and response could have significantly reduced the final number of dead waterfowl.

The presence and interaction of several risk factors associated with the multiplication of *C. botulinum* in the investigated aquatic environment likely contributed to the onset of the disease. The shallow depth of the lagoon, combined with high environmental temperatures, probably caused the water temperature to rise, leading to the optimal growth conditions (25–40 °C) for *C. botulinum*. Furthermore, an alkaline pH has also been identified as a requirement for bacterial growth, particularly when it is combined with high temperatures and low oxygen [15]. During the outbreak investigation, a pH of 9 was measured in the lagoon water. Although no previous pH data were available for comparison, this value suggests that the water pH provided conditions suitable for bacterial proliferation [15, 18]. However, given the lack of longitudinal data, the contribution of pH to the outbreak must be interpreted with caution. At the moment of the outbreak investigation, the presence of green algae on the lagoon surface was noted. Although it was not quantified, algal growth is known to contribute to oxygen depletion in stagnant waters, creating anaerobic conditions favorable to *C. botulinum* proliferation [2]. The cultivated soil surrounding the lagoon could still be considered a potential source of environmental contamination, as modified soils have been shown to have a higher overall prevalence of *C. botulinum* spores in some regions. Although in Argentina, BoNT type C or D was not reported, *C. botulinum* spores were found in 27% of non-virgin soils versus 16.3% of virgin soils, suggesting that human activities may influence the presence of the bacteria in the environment [19].

The waterfowl species, such as yellow-billed pintail and black-necked swan, might have been particularly affected over the course of the outbreak due to their significant presence in the lagoon and their higher susceptibility to BoNT type C toxin compared with other species [15]. The fact that no carcasses of other bird species, such as the Chilean flamingo or great egret, were found in the lagoon may be attributed to two possible factors. First, these bird species were less numerous in the lagoon than the affected waterfowl. Secondly, their dietary habits, which mainly include live prey and fish, result in a lower exposure to BoNT type C toxins. These observations align with another study, which reported that flamingos were also less vulnerable during outbreaks due to differences in feeding behaviour and habitat use [20]. Furthermore, species such as raptors and songbirds were not affected by the outbreak either. These groups of birds, particularly diurnal and nocturnal raptors, are less predisposed to the disease due to their limited contact with aquatic environments. The authors also hypothesize that certain birds may harbor specific gut microbiota that competitively inhibit BoNT-producing strains, potentially contributing to increased resistance [15].

The clinical signs of avian botulism, such as flaccid paralysis and limber neck, are characteristic but not specific, as they can be registered in other neurological diseases affecting birds [1]. Therefore, laboratory testing is essential to confirm botulism and differentiate it from other diseases, such as avian influenza [21]. This differentiation is especially important given the recent detection of highly pathogenic avian influenza virus (HPAIV) in Argentina, introduced by migratory wild birds [22]. At the time of this case, HPAIV had not yet been detected in the region, so samples were initially tested for botulism. This situation underscores the critical need for early detection and ongoing surveillance of avian diseases, particularly in the context of emerging diseases.

Although *C. botulinum* was found in the water, its presence in an aquatic environment can be a common finding and is not sufficient to confirm the origin of the outbreak [4]. However, bacteria identification can contribute to the diagnostic investigation by highlighting an increased risk of an outbreak in the investigated aquatic environment. To confirm botulism as a cause of death, the process must include field observations of clinical signs in animals, and epidemiologic conditions such as environmental factor and carcass presence, alongside with the detection of BoNTs in samples from affected animals [23].

Type C BoNT mainly affects birds but has also been reported in livestock [24]. In Argentina, these species often utilize lagoons as a water source, increasing the risk of exposure. In this specific case, livestock did not have access to the lagoon. However, it is recommended to avoid access to such water sources if botulism is suspected.

Although type C BoNT is not commonly associated with humans, concerns have been raised regarding the consumption of wild bird meat. In one study, genes encoding BoNT type E, known for its zoonotic potential, were detected in hunted birds [25]. Despite BoNT type E has not been reported in birds in Argentina to date, the detection in other regions suggests that environmental surveillance efforts for zoonotic BoNT should be considered in ecosystems where outbreaks occur, and where the consumption of meat from wildlife is common.

In free-ranging populations such as wild birds, vaccination is difficult to implement. Therefore, other management practices are essential to controlling the spread of botulism. Carcass removal is one of the most effective strategies for managing wild bird botulism, as it helps disrupt the carcass-maggot cycle, which sustains *C. botulinum* in the ecosystem [1, 15]. Invertebrates, particularly maggots, can accumulate BoNTs and *C. botulinum* when feeding on decomposing tissue. If healthy birds consume these contaminated invertebrates, they can become infected, further perpetuating the disease [26]. The observed reduction in bird mortality following carcass removal highlights the effectiveness of this intervention in limiting the progression of the outbreak. By interrupting the carcass-maggot cycle, this intervention likely reduced the persistence and spread of *C. botulinum* in the environment. Although the concurrent drop in ambient temperature may have contributed, the strong temporal association between the intervention and the decrease in mortality suggests that carcass management was a key control factor. These observations support the recommendation of rapid carcass disposal in future outbreaks, especially in ecosystems without formal wildlife health protocols. Due to the significant effectiveness of carcass removal in controlling botulism outbreaks and its complex epidemiology, the annual recording of environmental conditions, locations and dates can be relevant information for outbreak prevention. As a general recommendation, carcasses should be collected 15 days before expected risk periods and continued for 10–15 days after, to prevent the carcass-maggot cycle [27].

In conclusion, this first documented case of waterfowl botulism in the central region of Argentina underscores the need for epidemiological surveillance that enables early detection and prevention of further cases. This report also reinforces the importance of integrating environmental monitoring and community-based alert systems in wildlife disease management protocols.

## Supplementary Information

Below is the link to the electronic supplementary material.

Supplementary Material 1

## Data Availability

The datasets used and/or analyzed during the current study are available from the corresponding author on reasonable request.

## Declarations

### Prior publication

Data included in this article have previously been published in the Argentinian Association of Veterinary Laboratory Diagnosticians Congress, Mar Del Plata, Argentina, 11–13 September 2024.
